# ulfasQTL: an ultra-fast method of composite splicing QTL analysis

**DOI:** 10.1186/s12864-016-3258-1

**Published:** 2017-01-25

**Authors:** Qian Yang, Yue Hu, Jun Li, Xuegong Zhang

**Affiliations:** 1 MOE Key Laboratory of Bioinformatics and Bioinformatics Division, TNLIST, Department of Automation, Tsinghua University, Beijing, 100084 China; 2 Department of Applied and Computational Mathematics and Statistics, University of Notre Dame, Notre Dame, IN 46556 USA; 3 School of Life Sciences, Tsinghua University, Beijing, 100084 China; 4 Center for Synthetic and Systems Biology, Tsinghua University, Beijing, 100084 China

**Keywords:** Alternative splicing, Genetic variants, sQTL, Genome-wide, Ultra-fast method

## Abstract

**Background:**

Alternative splicing plays important roles in many regulatory processes and diseases in human. Many genetic variants contribute to phenotypic differences in gene expression and splicing that determine variations in human traits. Detecting genetic variants that affect splicing phenotypes is essential for understanding the functional impact of genetic variations on alternative splicing. For many situations, the key phenotype is the relative splicing ratios of alternative isoforms rather than the expression values of individual isoforms. Splicing quantitative trait loci (sQTL) analysis methods have been proposed for detecting associations of genetic variants with the vectors of isoform splicing ratios of genes. We call this task as composite sQTL analysis. Existing methods are computationally intensive and cannot scale up for whole genome analysis.

**Results:**

We developed an ultra-fast method named ulfasQTL for this task based on a previous method sQTLseekeR. It transforms tests of splicing ratios of multiple genes to a matrix form for efficient computation, and therefore can be applied for sQTL analysis at whole-genome scales at the speed thousands times faster than the existing method. We tested ulfasQTL on the data from the GEUVADIS project and compared it with an existing method.

**Conclusions:**

ulfasQTL is a very efficient tool for composite splicing QTL analysis and can be applied on whole-genome analysis with acceptable time.

## Background

The human genome contains about 3 billion base pairs, and there are only about 0.1% differences between two individuals’ genome [1]. These genetic variants largely contribute to human multiple phenotypes [2]. Genome-wide association studies (GWAS) have identified many genetic loci that are associated with diseases. Understanding how these variants exert their effects still remains to be a big challenge [3]. It has been observed that many of the effects are through variations in the expression of genes and pathways, especially RNA splicing [4].

Alternative splicing is an important mechanism in the regulation of gene expression. High-throughput RNA-sequencing (RNA-seq) data have shown that most human genes undergo alternative splicing [5, 6], and it has been reported that many alternative splicing events are associated with many complex diseases [7–10]. Expression quantitative trait loci (eQTL) analysis is an effective approach for studying the association between genetic variants and gene expression [11–16]. This strategy has been extended to the analysis of association of alternative splicing genes with genetic variants [15, 17–29]. This is called splicing quantitative trait loci (sQTL) analysis, including exon-level sQTL and isoform-level sQTL. For exon-level sQTL study, researchers take exon expression, exon inclusion level or junction expression as the quantitative phenotype to perform sQTL analysis against genetic variants [15, 17, 20, 23–26, 28, 29]. Exons in one gene are not independent and they compose multiple isoforms through alternative splicing. In some cases, changes in the splicing pattern of a gene cannot be observed by changes in inclusion levels of individual exons [27]. The expression of each individual isoform can also be used as the quantitative phenotype for sQTL study [18, 20, 21]. Coulombe-Huntington et al. [19], Lappalainen et al. [23] and Battle et al. [22] used the isoform ratio as the quantitative trait for sQTL analysis, which controls the effects of overall gene expression and tests the relative abundances of isoforms. But they took each isoform ratio as a phenotype and did not consider the correlations between isoforms of the same gene. In many situations, besides the expression of each isoform, compositions and relative proportions of alternative isoforms of the same gene play important roles. Monlong et al. [27] proposed to use the splicing ratios of all isoforms of the same gene as a composite phenotype to take into consideration such correlations. In this way, the studied phenotype is not only the relative abundance of each isoform but also the correlated structure of the alternative splicing gene. We call this as composite splicing QTL analysis. They developed an R package sQTLseekeR to implement this strategy, which describes alternative splicing events by a vector of splicing ratios [27]. They compared their method with other univariate sQTL methods that are based on exons or isoforms, and showed that sQTLseekeR is more capable of detecting associations that cannot be found by univariate exon-based method [27].

sQTLseekeR is based on tests on every gene-variant pair. Considering the tens of thousands genes and millions of genetic variants on the whole genome, the computational speed of sQTLseekeR prohibits it to be applied for analyzing all the genes and variants at the whole-genome scale. In their original work, they only applied it for analyzing variants located within 5 kb of each gene [27]. This largely limits the scope of questions that can be addressed with the method. Alternative splicing is regulated by both cis-elements and trans-factors [30]. More computationally efficient methods are in critical need for building the full picture of both cis- and trans-regulations of alternative splicing.

In this paper, we developed a method named ulfasQTL for ultra-fast composite sQTL analysis. It transforms vectors of splicing ratios to a spherical coordinate system and uses a matrix-based computation to test multiple genes and variants at the same time. This can dramatically boost the computational speed. We applied the proposed method and compared it with sQTLseekeR on data from the GEUVADIS project [23] to evaluate its performance and test its feasibility for genome-scale computation. Results show that ulfasQTL is several orders faster and can be readily used for genome-wide studies for associations between the alternative splicing structures of all genes and all variants in the genome.

## Methods

### Definition of splicing-QTL

Suppose a gene has *n* isoforms, and their expressions are *x*_1_, *x*_2_, …, *x*_*n*_. The splicing ratios of isoforms are their proportions in the total expression of the gene:

$$ {p}_i={x}_i/{\displaystyle {\sum}_1^n{x}_i},i=1,2,\dots, n. $$

Let a variant’s genotype be *g*, and *g* = 0, 1 or 2. Our goal is to detect associations between the genotype of a variant (the value of *g*) and the splicing pattern of a gene. For splicing pattern of a gene, we focus on the splicing ratios *p*_*i*_, *i* = 1, 2, …, *n* of the isoforms but not the gene’s total expression ∑_1_^*n*^ *x*_*i*_.

**Fig. 1:**
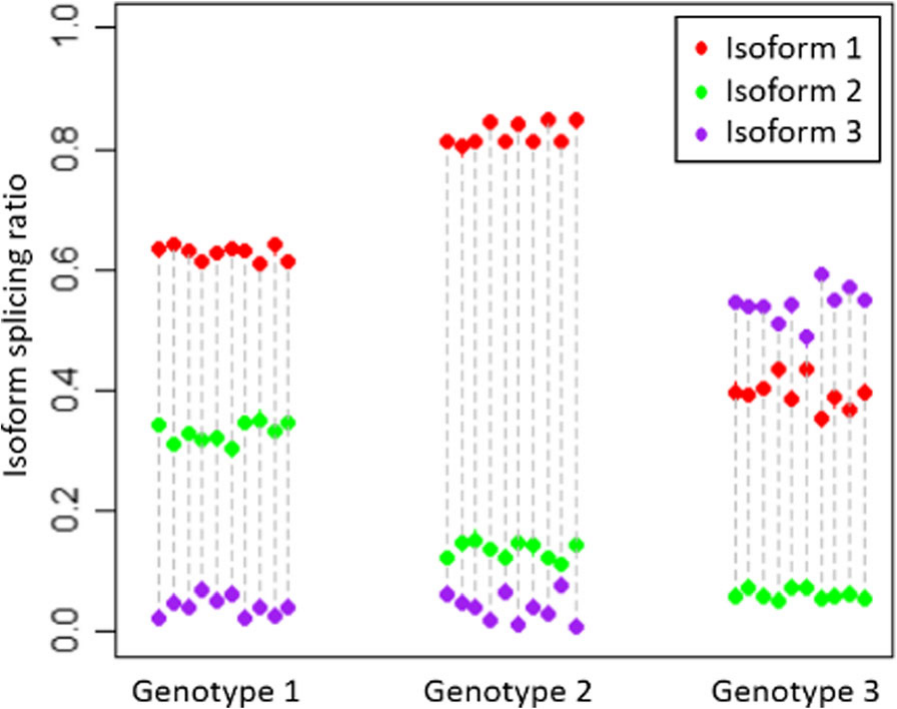
An example case of splicing-QTL. The gene has 3 isoforms. The splicing ratios of the three isoforms of the same sample are shown as points of different colors linked by a *dashed line*. Samples with the same genotype are shown together. We can see that the distribution patterns of splicing ratios are different between different genotypes, which indicates that this variant is associated with the alternative splicing pattern of this gene, and therefore it is a sQTL of the gene

The splicing pattern of a gene is described by the vector (*p*_1_, *p*_2_, …, *p*_*n*_). Figure 1 shows a simple example of a gene with 3 isoforms. The patterns of the splicing ratios are very different among samples of different genotypes of the variant, which indicates that the variant is a splicing-QTL or sQTL of the gene.

### The sQTLseekeR method

The sQTLseekeR method by Monlong et al. [27] uses a distance-based approach to detect composite sQTLs for each gene-variant pair. For one gene, each sample’s phenotype is the vector of the splicing ratios of all its isoforms. So each sample can be treated as a point in this vector space. All samples of a dataset are divided into the three or two groups according to their genotypes at a variant locus. sQTLseekeR calculates the variability of splicing ratios of a gene between and within groups using the Hellinger distance. For a gene containing *n* isoforms, the Hellinger distance between sample *a* and *b* is defined as

$$ {d}_H\left(a,b\right)=\sqrt{{\displaystyle {\sum}_{i=1}^n{\left(\sqrt{p_{ia}}-\sqrt{p_{ib}}\right)}^2}}, $$

where *p*_*ia*_ is the splicing ratio of isoform *i* in sample *a*, and *p*_*ib*_ is the splicing ratio of isoform *i* in sample *b*. The variability is defined as the sum of squared distances (*SS*) between the samples and their centroid,

$$ SS={\displaystyle {\sum}_{j=1}^N{d}_H^2\left(j,\boldsymbol{c}\right)}, $$

where ***c*** is the centroid, and *N* is the number of samples in this group. The within-group variability *SS*_*w*_ is defined as

$$ S{S}_W=SS={\displaystyle {\sum}_{j=1}^N{d}_H^2\left(j,\boldsymbol{c}\right).} $$

The between-group variability *SS*_*B*_ can be obtained by

$$ S{S}_B=S{S}_T-S{S}_W, $$

where *SS*_*T*_ is the total variability *SS*_*T*_ = ∑_*i* = 1_^*L*^ *d*_*H*_^2^(*c*_*i*_, ***c***), *L* is the number of variant’s genotype groups, *c*_*i*_ is the centroid of each genotype group, and ***c*** is the overall centroid of all samples.

The Anderson test [31] is used to compute a pseudo F-ratio score to measure the relative differences between within-group and between-group distances,

$$ F=\left[S{S}_B/\left(L-1\right)\right]/\left[S{S}_W/\left({\displaystyle {\sum}_{i=1}^L{N}_i-L}\right)\right], $$

where *L* is the number of groups and *N*_*i*_ is the number of samples in group *i*. They used a direct method to calculate the pseudo F-ratio score by considering matrix of distances between every pair of samples instead of using centroids in the definition [31]. The null distribution of the F-score is approximated via simulation to get the FDR (false discovery rate) of the tests.

Different genes contain different numbers of isoforms so their splicing ratio vectors are of different dimensions. Also different genetic variants divide samples with different grouping. Therefore, sQTLseekerR needs to test each gene against each variant individually. It is very time-consuming and infeasible for analyses at whole-genome scales.

### The ulfasQTL method

The goal of our ulfasQTL method is to detect composite sQTLs for all gene-variant pairs on the whole genome efficiently. The core strategy is to compute the statistics of associations for a large number of gene-variant pairs concurrently within a single run of the test. A matrix-based test for multiple independent phenotype-variant pairs is adopted to achieve the high computational efficiency, and we introduced a coordination transform on the splicing ratio vector to make the tests in the matrix independent. The test results on the ratios belonging to the same gene are then combined to produce the final statistics on the gene. We describe the details below.

Suppose there are *n* isoforms in a gene, and their splicing ratios are *p*_1_, *p*_2_, … *p*_*n*_, respectively. There is the constraint that ∑_*i* = 1_^*n*^ *p*_*i*_ = 1, and so the degrees of freedom of the vector (*p*_1_, *p*_2_, … *p*_*n*_) is *n* − 1. Thus, we cannot directly perform association analysis for all isoforms in a gene by adding the statistics up as the test for the gene because of their dependence. We need to transform the *n* splicing ratios to a set of *n-*1 independent variables. We propose to do this transformation using the idea of “n-sphere”. Firstly, let

$$ {q}_i=\sqrt{p_i},\kern0.5em i=1,2,\dots, n, $$

then one sample can be represented by the vector $\left({q}_1,{q}_2,\dots, {q}_{\mathrm{n}}\right)=\left(\sqrt{p_1},\sqrt{p_2},\dots, \sqrt{p_n}\right)$ in a *n*-dimensional Cartesian coordinate system. We convert it to coordinates in a spherical coordinate system (ρ, *ϕ*_1_, *ϕ*_2_, …, *ϕ*_*n* − 1_), where ρ is the length of the vector, defined as $\uprho =\sqrt{q_1^2+{q}_2^2+\dots +{q}_n^2}$, and *ϕ*_1_, *ϕ*_2_, …, *ϕ*_*n* − 1_ are the angles between the vector and *n*-1 of the Cartesian axes, defined as

$$ \begin{array}{l}{\phi}_1= arccos\frac{q_1}{\sqrt{q_1^2+{q}_2^2+\dots +{q}_n^2}},\\ {}{\phi}_2= arccos\frac{q_2}{\sqrt{q_2^2+{q}_3^2+\dots +{q}_n^2}},\\ {}\kern4em \dots, \\ {}{\phi}_{n-1}= arccos\frac{q_{n-1}}{\sqrt{q_{n-1}^2+{q}_n^2}}.\end{array} $$

In this way, the original *n* splicing ratios are converted to *n*-1 independent variables *ϕ*_1_, *ϕ*_2_, …, *ϕ*_*n* − 1_. We call them *converted splicing components* for convenience. The order of the *q*_1_, *q*_2_, …, *q*_*n*_ in the above transformation can be arbitrary. We order them from the largest to the smallest mean values across the samples in our implementation.

**Fig. 2:**
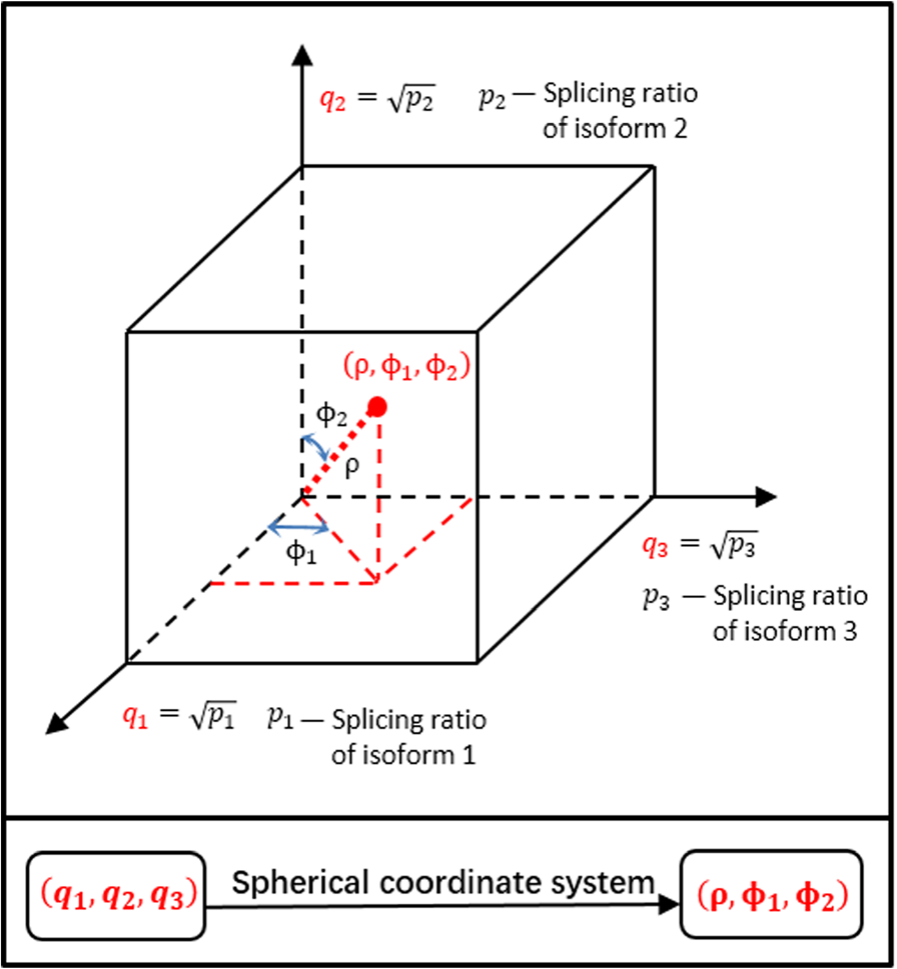
An illustrative example of spherical coordinate system application. The example gene contains 3 isoforms, and their splicing ratios in a sample are *p*_1_, *p*_2_, *p*_3_ with the constraint ∑_*i* = 1_^3^ *p*_*i*_ = 1. We let ${q}_i=\sqrt{p_i},\ i=1,2,3$ and present this sample as a point (the *red point* in the plot) in the 3-dimensional rectangular coordinate system. We then apply spherical coordinate system in which this sample can also be regarded as a point (ρ, ϕ_1_, ϕ_2_) in a spherical coordinate system. Because of the constraint that ∑_*i* = 1_^3^ *p*_*i*_ = 1, the spherical coordinates of this sample is (1, ϕ_1_, ϕ_2_), in which ϕ_1_ and ϕ_2_ are independent from each other

Figure 2 illustrates how the spherical coordinate system works with an example gene. In the example, the gene contains three isoforms. The original constraint ∑_*i* = 1_^3^ *p*_*i*_ = 1 on the splicing ratios becomes

$$ {q}_1^2+{q}_2^2+{q}_3^2=1 $$

on the *q*_*i*_’s. In the spherical coordinate system, we always have ρ = 1 regardless of the values of *p*_1_, *p*_2_, and *p*_3_. The two angles in the spherical coordinate system, *ϕ*_1_ and *ϕ*_2_, on the other hand, are independent from each other.

In [32], Shabalin proposed a matrix-based method Matrix eQTL for fast eQTL computation. It can test all gene-variant pairs together by choosing appropriate test statistics and applying matrix operations to calculate their test statistics values in parallel. It implements both linear regression model and ANOVA model for eQTL analysis. Matrix eQTL can detect associations between two variables, but our goal is to detect associations between vectors and variables. After spherical coordinate transformation, we converted a vector into a set of mutually independent variables, and then adopted this matrix-based strategy in ulfasQTL to implement massive tests on the converted splicing components *ϕ*_*i*_ ’ *s* in a matrix. Suppose we want to do tests on *m* genes and *k* variants of *l* samples in a single run, the expression of these genes can be represented by a matrix *G*_m__*__l_ and the genotypes of the variants can be represented by a matrix *V*_k*__l_. Now we do the tests on the converted splicing components instead of the expression values. So we build the matrix Φ of all converted splicing components of the *m* genes. The dimension of this matrix is t*l, where *t* is the total number of independent splicing components of the *m* genes, which equals to the total number of isoforms of these genes minus the number of genes. The columns (samples) of the matrix Φ_t*__l_ and matrix *V*_k*__l_ are matched with each other.

Here is the detailed method of Matrix eQTL for linear regression model, and the method of Matrix eQTL for ANOVA model is similar to linear regression model [32]. We assumed that the association between splicing component *ϕ* and variant *v* is linear.

$$ \phi =\alpha +\beta v+\epsilon, \kern0.75em \mathrm{where}\ \epsilon \sim \mathrm{i}.\mathrm{i}.\mathrm{d}.\ \mathrm{N}\left(0,{\sigma}^2\right) $$

For linear regression model, Matrix eQTL chose the absolute value of sample correlation |*r*| = |cor(*ϕ*, *v*)| as the test statistic which can has equal power and can be computed faster than other test statistics. Then Matrix eQTL performed standardization preprocessing procedures which do not change the correlation.

$$ {\displaystyle \sum }{\phi}_i=0,{\displaystyle \sum }{\phi}_i^2=1,{\displaystyle \sum }{v}_i=0,{\displaystyle \sum }{v}_i^2=1 $$

So Matrix eQTL computed the test statistics by the inner product 〈*g*, *v*〉 between vectors *ϕ* and *v* as follows.

$$ {r}_{gv}=\mathrm{c}\mathrm{o}\mathrm{r}\left(g,v\right)=\frac{{\displaystyle \sum}\left({g}_i-\overline{g}\right)\left({v}_i-\overline{v}\right)}{\sqrt{{\displaystyle \sum }{\left({g}_i-\overline{g}\right)}^2{\left({v}_i-\overline{v}\right)}^2}}={\displaystyle \sum }{g}_i{v}_i=\left\langle g,v\right\rangle $$

Matrix eQTL can greatly simplify the computation of test statistics by the multiplication of the two preprocessed matrices Φ_*t* ∗ *l*_ ⋅ *V*_*k* ∗ *l*_^*T*^ [32]. In this way, the computational load can be reduced dramatically. The key assumption for this fast computation is the rows (components) in the matrix are independent with each other, which is guaranteed by the spherical coordinate transformation.

Matrix eQTL can conduct either linear regression or ANOVA based on the obtained correlations, and report the *t*-test statistics or *F*-test statistics of all associations between each converted splicing component and each variant. After getting all test statistics for each component-variant pair, we combine results from all components of the same gene-variant pair to get the test statistic for the gene-variant pair. We convert *t*-test statistics or *F*-test statistics of the component-variant pairs to *z* values that follow the standard normal distribution. Finally, we get the test statistic *s* for each gene-variant pair by

$$ s={\displaystyle {\sum}_{i=1}^{n-1}{z}_i^2}. $$

**Fig. 3:**
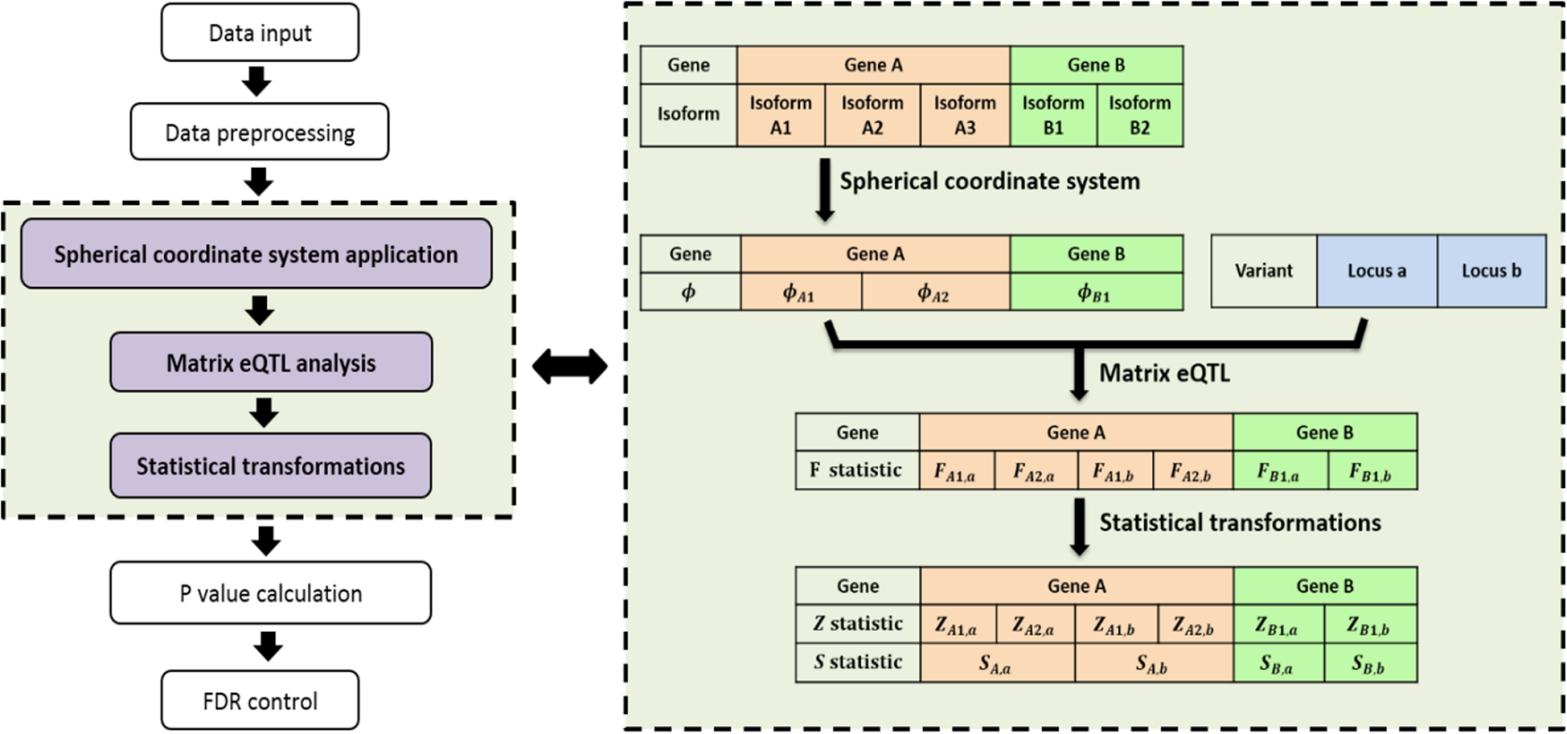
The diagram of ulfasQTL. The left panel is basic flowchart and the right panel shows an illustrative example of core steps. There are in seven steps in the flowchart and the 3 steps shown in purple are core steps of ulfasQTL. We have two matrices after data preprocessing. One for the genotype data (genotypes of each sample at loci a and b) and one for the isoform expression data (isoforms A1, A2, A3 of gene A and isoforms B1, B2 of gene B of each sample). Then isoform expression matrix is converted to splicing components matrix (*ϕ*_*A*1_, *ϕ*_*A*2_, *ϕ*_*B*1_ for each sample) by the spherical coordinate system. We then send genotype data and splicing components matrix into MatrixeQTL (ANOVA model) to get the *F* statistics (*F*_*A1,a*_, *F*_*A2,a*_, *F*_*A1,b*_, *F*_*A2,b*_, *F*_*B1,a*_, *F*_*B2,b*_) for each component-variant pair. Finally, we apply statistical transformations and get corresponding *Z* statistics according to *F* statistics, and merge *Z* statistics into *S* statistics (*S*_*A,a*_, *S*_*A,b*_, *S*_*B,a*_, *S*_*B,b*_) for each gene-variant pair

It follows a chi-square distribution with degrees of freedom *n-*1, i.e., *s* ~ *χ*_*n* − 1_^2^, and the *p*-value for each gene-variant pair can be obtained accordingly. We can then convert the *p*-values to false discovery rates (FDRs) using the q-value method [33]. We developed a software package ulfasQTL to implement the above method, which calls for the MatrixEQTL package [32] in the matrix calculation. Figure 3 shows the basic flowchart of the whole method (the left panel) and a detailed illustrative example (the right panel).

When applying the method on very large datasets like genome-wide analyses, the dataset can be too large to be fit into computer memory. In such cases, we split dataset into smaller subsets and calculate them in multiple runs. For example, in the experiments reported below, we take all genes together but split the variants into smaller files, each containing 1000 variants.

The ulfasQTL package was developed using R and C++. It includes C++ codes for data preprocessing and the spherical coordinate transformation, and R codes for Matrix eQTL analysis and the calculation of *p*-values and FDRs. The package can be downloaded at http://bioinfo.au.tsinghua.edu.cn/software/ulfasQTL/.

### The computational complexity

The computing time of ulfasQTL is consumed mostly by two major steps. One is the computation with MatrixEQTL for calculating the correlation matrix of components and variants. The dimension of component matrix is t*l, the dimension of variant matrix is k*l. So the time complexity of this step is O(k*t*l), where *k* is the total number of variants, *t* is the total number of converted splicing components and *l* is the total number of samples. The second major step is that after getting the MatrixEQTL output, we need to sort the component-variant pairs by both splicing components and variants to make sure pairs from the same gene and the same variant stay together. We need to sort the pairs twice for that purpose. We use the mergesort method as it is one of the fastest stable sorting method. The time complexity of mergesort is O(t*k*log(t*k)). Therefore, the total time complexity of ulfasQTL is *O((l +* log(t*k))*t*k).

For large datasets that need to be split into multiple smaller datasets, the computation on the multiple datasets can be assigned to multiple kernels or computers, which provides an easy and efficient way of doing large-scale sQTL analysis in parallel.

For each gene-variant pair, sQTLseekeR calculates the within-group variability and the between-group variability to get the Anderson test statistic for the pair. The complexity for this step is *O(l* ^*2*^ *)*. The test method used by sQTLseekeR is sensitive to the homogeneity of the variabilities or dispersions of the compared groups. The test power may decrease when dispersions of the groups are very different. So sQTLseekeR needs an extra step to filter such variants to avoid potential false sQTLs. The method for this filtering is similar to ANOVA, but the distance measurement is different from Euclidan distance. They applied principle component analysis (PCA) to the data and calculated the Euclidan distances between group members and the group centroid on the principal components. The time complexity of computing the eigenvalue in PCA of a l*l dimensional matrix is *O(l* ^*3*^ *)*, and computing the within-group variability is *O(l* ^*2*^ *)*. For all phenotype-variant pairs of *m* genes and *k* variants, the total time complexity of the above steps is *O(l* ^*3*^ *m*k). After getting the *F* score of a candidate pair and this filtering step, sQTLseekeR performs an approximation of permutations for each gene to calculate the significance of the *F* score. The computational complexity of this step is *O(l* ^3^*m*)*. So the overall complexity of sQTLseekeR is at the level of *O(l* ^*3*^ *m*k).

## Results

### Data

We applied ulfasQTL on the data of lymphoblastoid cell lines of 462 individuals published in [23] to study its performance. The transcripts expression data are from the GEUVADIS project [23] and the genotype data are from 1000 Genomes Project Phase I dataset 1 [1]. The dataset includes individuals from European population (CEU, FIN, GBR, TSI) or African population (YRI). For isoform expression data, at first we added a small number to the expression data to avoid the occurrence of 0’s in the denominator. Next we computed the splicing ratios of each isoform of all genes, and only considered active isoforms with splicing ratios larger than a given threshold. Genes with less than two active isoforms after this step were filtered out. Then we calculated the splicing variability for each gene and removed genes whose splicing variability are less than 0.01. For each gene, we used samples whose gene expression is over 0.01 RPKM. For genotype data, we kept variants that have at least 2 genotype groups in the samples and each group has at least 5 samples. Groups with less than 5 samples are set to *NA* to make sure that they are not taken into consideration in the test. We picked up samples which have both expression data and genotype data, and made the samples’ order identical in two data files.

We conducted 3 experiments, Experiments I, II and III. Experiments I and II were on small-scale datasets to study the performance of ulfasQTL and to compare it with sQTLseekeR. Experiments III was on a genome-scale dataset to test the feasibility of ulfasQTL on big data. The experiments were done on a desktop computer with CPU of Intel Core i7-4790 k(4GHz) and 16GB DDR3 RAM, running 64 bit Ubuntu and 64 bit R 3.2.3.

### The computational efficiency

Experiments I and II were on a small dataset on which both ulfasQTL and sQTLseekeR can work. In Experiment I, we randomly picked 1000 variants and 407 genes containing a total of 1000 isoforms in Chr.1. We performed sQTL analysis using both methods to compare the computational efficiency and results of the two methods. It took 13,680 s (3.8 h) for sQTLseekeR to complete the computation, while the ulfasQTL only used 2.3 s to complete the computation. ulfasQTL works about 6000 times faster than sQTLseekeR.

In Experiment II, we choose 400 genes and 180,446 variants which are all located at 1–13,000,000 in Chr.1. After preprocessing on the expression data and variant data, 160 genes and 76,779 variants were kept, which gave 12,284,640 candidate gene-variant pairs. ulfasQTL accomplished all the computation in 901 s (15.0 min). We applied sQTLseekeR on these 160 genes with only the variants that are located within 5 kb of each gene as in the original work. This gave a total of 8560 candidate gene-variant pairs. sQTLseekR used 4492 s (~1.25 h) to complete these computations.

Experiment III was on all genes and genetic variants on Chr.1 to test the feasibility of ulfasQTL for genome-scale analyses. There are in total 5172 genes and 1,900,188 variants after screening. The total number of gene-variant pairs which need to be tested are 9.8x10^9^. On the same desktop computer as in the first experiment, ulfasQTL can give the result of a split subset of 5172 genes and 1000 variants in about 45 s. The analysis on the whole task took 87,112 s (~24.20 h).

Applying sQTLseekeR on the data of Experiment III is impractical due to the heavy computing cost. In the original sQTLseekeR paper [27], the authors reported that they ran sQTLseekeR separately in each sub-population on this dataset, and each sub-population contains about 10,012 genes and 140 variants per gene on average. The analysis of ~1,400,000 gene-variant pairs took about 4 h using 16 cores (2Gb 2.70GHz nodes). Based on these reports, we can estimate that it would take about 1169 days or 3.2 years on a similar cluster if sQTLseekeR were to be used to analyze the data in Experiment III.

### Comparison of *p*-values

**Fig. 4:**
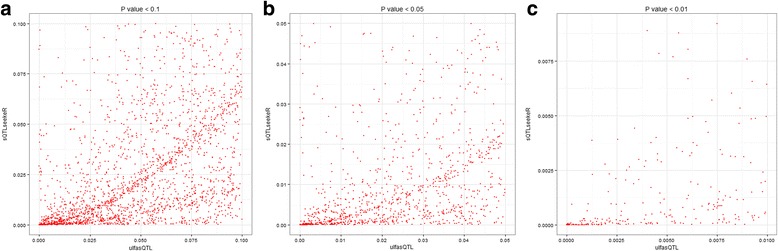
*P*-values of the two methods on the same points (candidate associations) in the range of *p* < 0.1, *p* < 0.05 and *p* < 0.01 in Experiment I. The *horizontal axes* are *p*-values of associations detected by ulfasQTL and the *vertical axes* are *p*-values of associations detected by sQTLseekeR

We compared the results of ulfasQTL and sQTLseekeR in Experiments I and II to obtain better understanding on the similarities and differences between the tests used by the two methods. In Experiment I, after data preprocessing there were 359 candidate variants and 140 candidate genes that were analyzed by both ulfasQTL and sQTlseekeR. They composed 50,260 candidate associations to be tested by ulfasQTL and sQTLseekeR. sQTLseekeR adopted some further filtering on the genes and only tested 47,069 of the candidate associations. We used these 47,069 candidate associations to study the relationship of *p*-values reported by the two methods. Figure 4 shows the scatter plots of the *p*-values of the two methods on the same points (candidate associations) in the range of *p* < 0.1, *p* < 0.05 and *p* < 0.01. The Spearman correlations of the two *p*-values are 0.58, 0.56 and 0.73, respectively, for candidate associations with *p*-values less than 0.1, 0.05 and 0.01. We can observe that ulfasQTL tends to be more conservative and tends to produce slightly larger *p*-values for most of the data. Note that the data in this experiment were a randomly selected subset of genes and SNPs on Chr.1. We can expect that most of the candidates would not be significant. The more conservative *p*-value obtained with ulfasQTL presents an advantage over existing method not only on the higher computational efficiency, but also on the possible lower false discoveries.

**Fig. 5:**
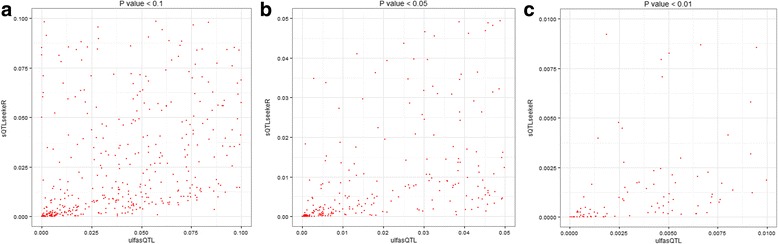
*P*-values of the two methods on the same points (candidate associations) in the range of *p* < 0.1, *p* < 0.05 and *p* < 0.01 in Experiment II. The *horizontal axes* are *p*-values of associations detected by ulfasQTL and the *vertical axes* are *p*-values of associations detected by sQTLseekeR

In Experiment II, 160 candidate genes and 76,779 candidate variants were analyzed by ulfasQTL, and 160 candidate genes and variants located within 5 kb from them were analyzed by sQTLseekeR. After preprocessing, we got a total of 8560 candidate associations that have *p*-values reported by both methods. Figure 5 shows the scatter plots of the *p*-values of the two methods on the same points in the range of *p* < 0.1, *p* < 0.05 and *p* < 0.01. The Spearman correlations of the two *p*-values are 0.60, 0.69 and 0.67, respectively, for candidate associations with *p*-values less than 0.1, 0.05 and 0.01. We can see that the general trends of relations of the *p*-values are the same in Experiments I and II, while the correlation between the results of the two methods is higher in Experiment II. Experiment I was on randomly selected genes and variants so it can be expected that most of the gene-variants pairs are not significantly associated. On the other hand, candidate variants compared in Fig. 5 in Experiment II were all within 5 kb of the candidate genes, which are more likely to have significant sQTLs. The higher correlation between *p*-values of the two methods implies that the two methods agrees better with each other on true association signals.

## Discussion

There are several directions that need further investigation. We used ANOVA to test the hypothesis in the method based on two underlying assumptions. The first one is the distribution of data should be normal distribution or close to normal distribution. We can see that the distribution of converted splicing components may not always meet the assumption. The other one is ANOVA assumes homogeneity among groups, which may be violated when the sample size of one group is small. Such situations can cause false positives. The preprocessing to add a small value to the denominator also may cause false results for some special cases when all isoforms are not expressed in some samples. Therefore, after applying ulfasQTL on genome-wide candidates, users may use slower single-gene based methods only on the reported results to further validate the significance if necessary, or to check homogeneity (such as using Bartlett’s test) of different genotype groups.

Composite splicing QTL involves the collaborative regulation of multiple isoforms. Comparing to the traditional univariate isoform- or exon-based splicing QTL analysis, golden-standard validation data is less available. Monlong et al. [27] illustrated a few examples of composite splicing QTLs, but due to the small scale of their work, the examples cannot be taken as standard. Actually, when applied on a larger range of candidate variations with sQTLseekeR on fewer genes, we observed that some examples became no longer significant after multiple test correction. This may be due to the nature that splicing composite variation is associated by the multiple genetic factors. The ability to conduct genome-wide study of composite sQTL by ulfasQTL can help to better investigate both cis- and trans- factors that can be associated with splicing composite variation, and it will be of great interest if methods can be developed for finding associations of composite splicing phenotypes with multiple genomic variation loci.

## Conclusions

We developed a new method ulfasQTL for ultra-fast splicing QTLs analysis of splicing patterns that are associated with genetic variants. This is the first time that coordination conversion is used for decomposing composite splicing pattern to a set of independent components. This conversion allows for the simultaneous computation on many genes in a matrix form. Experiments on small- and large-scale data show that it is several thousand times faster than the existing method for splicing QTL, and is efficient for splicing QTL analysis at the whole-genome scale.
